# Prevalence of Celiac Disease in China Among High‐Risk Populations: A Systematic Review and Meta‐Analysis

**DOI:** 10.1111/1751-2980.70013

**Published:** 2025-11-03

**Authors:** Ying Lian Xiao, Ya Qi Jia, Li Qun Gu, Min Hu Chen

**Affiliations:** ^1^ Department of Gastroenterology The First Affiliated Hospital of Sun Yat‐Sen University Guangzhou Guangdong Province China; ^2^ Medical Affairs, Takeda (China) Holdings Co., Ltd Beijing China; ^3^ Takeda Development Center Asia Shanghai China

**Keywords:** celiac disease, China, irritable bowel syndrome, prevalence, type 1 diabetes mellitus

## Abstract

**Objective:**

We conducted this systematic review and meta‐analysis to summarize the prevalence of celiac disease (CeD) among high‐risk populations in China.

**Methods:**

A systematic search was conducted in PubMed, EMBASE, Cochrane Library, Web of Science, and four Chinese databases to identify studies published up to December 10, 2024 on the prevalence of CeD in different regions of China. The high‐risk populations included patients with irritable bowel syndrome, gastrointestinal symptoms, autoimmune disease, low body mass index, short stature, etc. Data were extracted from the included studies by two reviewers independently using a standardized data extraction form. Regional prevalence estimates were weighted using China Population Census Yearbook 2020 data. The pooled prevalence of CeD was summarized using random‐effects models.

**Results:**

Twenty‐eight studies involving 9531 individuals were included. Biopsy‐confirmed prevalence and seroprevalence varied widely by region. Central China had the highest biopsy‐confirmed prevalence of CeD in high‐risk populations (4.55%, 95% confidence interval [CI] 0.66%–8.44%), followed by North China (3.60%, 95% CI 1.75%–6.08%) and East China (2.50%, 95% CI 1.39%–3.94%). Northeast China led in seroprevalence (7.10%, 95% CI 1.14%–13.05%), followed by Central China (4.42%, 95% CI 1.04%–9.98%), East China (4.37%, 95% CI 2.12%–7.38%), and North China (4.35%, 95% CI 2.39%–6.86%). The biopsy‐confirmed prevalence and seroprevalence of CeD among high‐risk populations in China were 3.69% and 4.43%, respectively.

**Conclusion:**

CeD prevalence appears to be significant among high‐risk populations and varies by geographical regions in China.

## Graphical abstract

Celiac disease (CeD) is an immune‐mediated condition with increasing recognition in China, but its actual prevalence, especially among high‐risk populations, remains unclear. This study provides the first pooled prevalence estimates of CeD among high‐risk populations in China, revealing significant regional variations in both biopsy‐confirmed prevalence and seroprevalence. EMA, endomysial antibody; tTG, tissue transglutaminase.

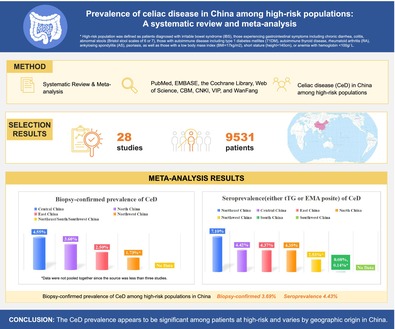

## 1 Introduction

Celiac disease (CeD) is an immune‐mediated disorder triggered by gluten ingestion in genetically predisposed individuals [1]. CeD exhibits a wide array of manifestations in both gastrointestinal (GI) and extraintestinal forms [2]. This clinical heterogeneity poses challenges for timely diagnosis of the disease, contributing to its global underdiagnosis [3]. Undiagnosed or untreated CeD can lead to severe complications and long‐term consequences, such as osteoporosis, neurological disorders, infertility, and an increased risk of intestinal malignancy [4, 5]. Currently, lifelong adherence to a strict gluten‐free diet represents the sole treatment for CeD. However, gluten avoidance poses significant challenges, with a treatment burden of the disease ranking second only to end‐stage renal disease [6]. Moreover, CeD entails direct medical costs and productivity losses [7].

Diagnosis of CeD typically involves serological tests, such as immunoglobulin A anti‐tissue transglutaminase antibody (anti‐tTG‐IgA) and anti‐endomysial antibody (EMA), along with duodenal mucosal biopsy, which serves as the gold standard [8, 9]. A previous study on the prevalence of CeD conducted in European countries has revealed that CeD affects about 1% of the population [10]. Other regions with high European ancestry, such as North America [11], Latin America [12], and Western Australia [13], have also reported its high prevalence. According to a recent systematic review and meta‐analysis, the global prevalence of CeD was 0.7% based on biopsy findings and 1.4% based on serological test results, respectively, with Asia reporting the highest seroprevalence (1.8%) while Africa having the lowest seroprevalence (1.1%) [3]. In 2021, Zhou et al. reported a CeD seroprevalence of 0.96% among the adult population and 0.11% in pediatric individuals In China [14]. Studies have revealed that the seroprevalence of CeD in Chinese populations varies according to the geographical regions [14, 15]. In addition, an underlying diseases can also affect the prevalence of CeD.

Identifying and focusing on high‐risk populations for CeD is crucial for the improvement of the diagnostic yield and guidance of effective public health strategies. Based on the 2013 American College of Gastroenterology (ACG) Clinical Guideline [16], individuals at an elevated risk for CeD include first‐degree relatives of the patient, those with autoimmune conditions (such as type 1 diabetes mellitus [T1DM] and autoimmune thyroid disease [AITD]), genetic syndromes like Down's syndrome or Turner's syndrome, unexplained iron deficiency anemia, osteoporosis, or persistent GI symptoms unresponsive to standard treatments. For example, the prevalence of CeD among individuals with irritable bowel syndrome (IBS) was 1.01%–2.85% [17, 18], whereas its seropositivity rate in patients with GI diseases, such as chronic diarrhea, recurrent GI hemorrhage, abdominal pain, tumor, or constipation, has been reported to be 13.97% [19]. Despite the recognized higher prevalence of CeD in these high‐risk groups, there remains a significant unmet need due to a delayed diagnosis, limited implementation of targeted screening protocols, and lack of patient and clinician awareness, particularly in regions with constrained healthcare resources.

In China, the awareness of CeD has been increasing during the past decades, leading to a surge in research in this field [20, 21, 22]. However, there have been insufficient nationwide epidemiological studies, with most studies being retrospective, small‐scale, regional‐based, Chinese‐language articles, which have limited their generalizability and international visibility. To better understand the status of CeD in China, we conducted a systematic review and meta‐analysis to estimate the biopsy‐confirmed prevalence and seroprevalence of CeD among high‐risk populations across China.

## 2 Materials and Methods

This study was conducted following the Preferred Reporting Items for Systematic reviews and Meta‐Analyses (PRISMA) 2020 statement [23]. The study protocol was registered in the PROSPERO database (https://www.crd.york.ac.uk/PROSPERO/; no. CRD42023451245).

### 2.1 Data Sources and Search Strategy

To identify all studies on the seroprevalence and/or biopsy‐confirmed prevalence of CeD among individuals in China, we conducted a literature search of PubMed, EMBASE, Cochrane Library, Web of Science, Chinese Biomedical Database (CBM), China National Knowledge Infrastructure (CNKI), VIP's Chinese Science and Technology Journal Database (VIP), and Wanfang Digital Resource Database (Wanfang), covering all articles published from database inception till December 10, 2024, without restrictions on language, publishing date, or publication status. Two authors independently conducted the literature search. The search terms and search strategies are detailed in Table S1.

### 2.2 Inclusion and Exclusion Criteria

The inclusion criteria of the studies included in the systematic review and meta‐analysis were as follows: (i) studies that evaluated the prevalence of CeD in high‐risk populations in China, including patients with IBS, experiencing GI symptoms and signs such as chronic diarrhea, colitis, or abnormal stool (Bristol Stool Scale of 6 or 7), with autoimmune disease such as T1DM, AITD, rheumatoid arthritis (RA), ankylosing spondylitis (AS), and psoriasis, and those with a low body mass index (BMI) (< 17 kg/m^2^), short stature (height < 140 cm), or other conditions such as anemia (hemoglobin [Hb] < 100 g/L) [14, 16, 24, 25, 26]; (ii) studies that focused on biopsy‐confirmed (defined by the original studies) or seroprevalence of CeD (i.e., tested by anti‐tTG‐IgA or EMA) across different regions of China. When calculating the overall seropositivity rate in high‐risk populations, we first selected the tTG testing results. If only EMA results were available, we used them for analysis. The regions of China were divided into Central, East, North, South, Southwest, Northwest, and Northeast [27]; and (iii) studies with a design limited to prevalence.

We excluded studies that solely reported antigliadin antibody (AGA) results because this test is not recommended for use in CeD diagnostic algorithms [28]. In addition, we excluded literature reviews, meta‐analyses, guidelines or consensus, animal studies, case reports, or case series from our analysis. We also excluded studies with a sample size of < 10 cases. Those with completely identical text were excluded as duplicates. Two authors screened the titles and abstracts of the search results independently. All potentially relevant citations were requested and inspected in detail using the full‐text version. For duplicates, studies with the largest sample size or most recent data were included for analysis. In addition, if two articles reported identical outcome data, the dataset from the earlier publication year was selected. Any disagreement was resolved by discussion, with the assistance of a third author. We contacted the authors of the identified articles to obtain the relevant information when there was insufficient data or doubt about the publications.

### 2.3 Data Extraction

The following information was extracted from each study: the first author's name, year of publication, region of the population studied, study design, study size and number of cases, participants' age and gender, diagnostic approach for CeD, and underlying high‐risk conditions. Data extraction was conducted by two authors independently using a standardized data extraction form. Any disagreement was resolved by consensus or discussion with a third author.

### 2.4 Quality Assessment

The risk of bias of the included studies was assessed by two authors independently. Prevalence studies were assessed using the Joanna Briggs Institute (JBI) critical appraisal checklist for prevalence studies [29]. Any discrepancy or inconsistency was resolved by discussion with a third author.

### 2.5 Statistical Analysis

R Statistical Software version 4.1.1 (R Foundation, Vienna, Austria) was employed to conduct the meta‐analysis using random‐effects models. For variables available from less than three studies, a narrative description was provided instead. For dichotomous outcomes, we extracted the reported rates and calculated the proportion and the corresponding 95% confidence interval (CI) for each study, and then pooled them by meta‐analyses. Range was used to describe the age of the included patients at baseline, and numbers and proportions were employed for other baseline factors.

We first estimated the prevalence of CeD among the high‐risk populations across different regions of China by meta‐analysis using a random‐effects model. Because of the differences in ethnic groups, dietary habits, and sizes of the populations across these regions, which could have potentially affected the prevalence of CeD, we estimated the adjusted prevalence of CeD in the high‐risk populations as follows. First, we combined the previously pooled prevalence in seven regions of China with their respective population sizes based on the China Population Census Yearbook 2020 [30] using Equation (1), where $a_{i}$ represents the prevalence of CeD in area *i* and $b_{i}$ represented the population proportion in area *i*:

(1) $$f\left(x\right)=\frac{\sum_{1}^{i}\left(a_{i}b_{i}\right)}{\sum_{1}^{i}b_{i}}$$

A meta‐analysis was then performed based on different risk factors among CeD patients, specifically the diseases or conditions they had, to estimate the CeD prevalence.

To determine the seroprevalence of CeD, we used anti‐tTG‐IgA results as the primary measure. In cases where anti‐tTG‐IgA data were unavailable, we relied on the EMA results. However, when a study provided both anti‐tTG‐IgA and EMA results, we gave priority to the anti‐tTG‐IgA results in our analysis.

Several analyses were performed to evaluate the heterogeneity of the included studies. The *I* ^2^ statistic over 50% and a significant *χ* ^2^ statistic indicated substantial heterogeneity [31]. Furthermore, sources of heterogeneity based on gender, age (children vs. adults), ethnicity, and region were assessed. If significant heterogeneity was present and the source could not be identified, we used a random‐effects model with restricted maximum likelihood (REML)‐based estimation of between‐study variance (*τ* ^2^) to pool the data. Funnel plots were used to investigate publication bias concerning the prevalence of CeD among the high‐risk populations.

## 3 Results

**FIGURE 1:**
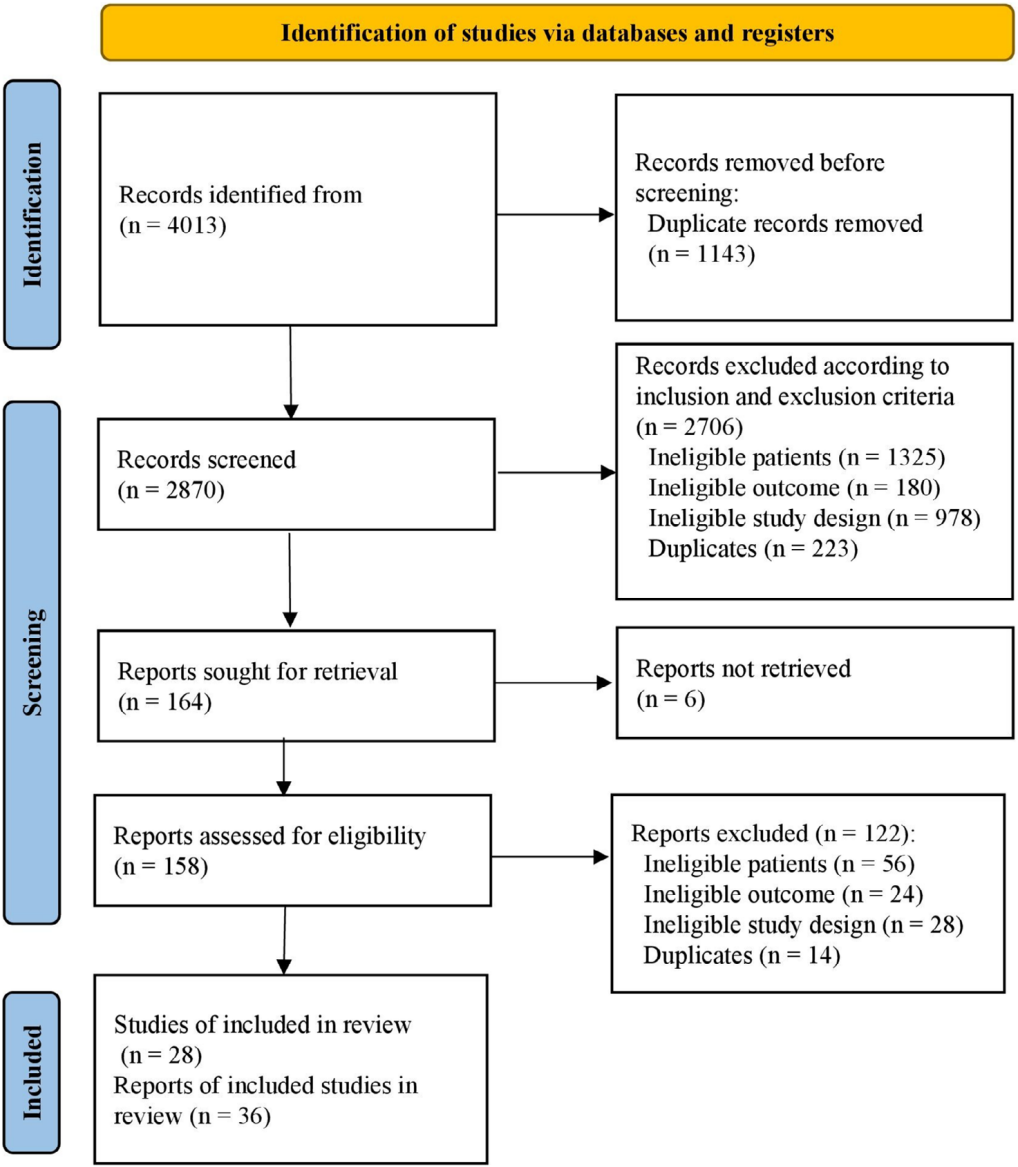
Flow diagram for identification of relevant studies.

During the initial search, 4013 records were identified, from which 1143 duplicates were excluded. Following the manual evaluation of titles and abstracts for relevance, 164 articles met the eligibility criteria and were selected for detailed evaluation. After further excluding 6 articles which could not be obtained due to unauthorized access, the remaining 158 articles were assessed for eligibility, from which 122 were excluded (Figure 1). Finally, a total of 28 studies (36 references) comprising 9531 Chinese individuals were included in this study [17, 18, 19, 32, 33, 34, 35, 36, 37, 38, 39, 40, 41, 42, 43, 44, 45, 46, 47, 48, 49, 50, 51, 52, 53, 54, 55, 56, 57, 58, 59, 60, 61, 62, 63, 64], of which 13 studies evaluated biopsy‐confirmed CeD and 26 studies evaluated its seroprevalence. Eleven studies appeared in multiple reports. Among all the included studies, 5 studies (8 references) provided information on IBS [17, 18, 39, 42, 43, 54, 55, 56], 7 on T1DM [33, 43, 46, 59, 60, 61, 64], 14 (20 references) on GI symptoms [19, 32, 33, 35, 36, 37, 38, 41, 43, 44, 45, 47, 48, 49, 50, 51, 52, 53, 62, 63], 1 on non‐segmental vitiligo [58], and 1 on abnormal liver function [57]. In addition, six studies [34, 35, 40, 43, 61, 64] offered data from multiple populations, including patients with colitis, diarrhea, anemia, low BMI, short stature, RA, AS, psoriasis, type 2 diabetes mellitus (T2DM), AITD, non‐autoimmune thyroid disease (NAITD), autoimmune polyglandular syndrome (APS) type 3 variant (APS3v), and so on, and those with a Bristol Stool Scale of 6 or 7. However, a meta‐analysis was not feasible for these populations because of insufficient data. Characteristics of all included studies are summarized in Table 1.

**TABLE 1:** Characteristics of the included studies.

First author (year of publication)	Regions of China	Province/Municipality/Autonomous Region	Diagnostic approach	High‐risk conditions	Sample size (*N*)	Age, years (range)	Male gender (*n*, %)	CeD (+)^c^ (*n*/*N*, %)	tTG‐antibody (+) (*n*/*N*, %) (overall/CeD)	EMA (+) (*n*/*N*, %) (overall/CeD)	AGA/DGP (+) (*n*/*N*, %) (overall/CeD)	Biopsy (+) (*n*, %)	Chinese‐/English‐language
Cao (2015) [32]	Central	Henan	tTG‐antibody, AGA, biopsy	GI symptoms (chronic diarrhea)	198	18–78	82 (41.41)	15/198 (7.58)	19/198 (9.60) 14/15 (93.33)		53/198 (26.77) 15/15 (100)	15/198 (7.58)	Chinese
Chen (2018) [33]	Northeast	Liaoning	EMA, AGA	T1DM^a^ or GI^a^ symptoms (diarrhea, abdominal distension or pain, vomiting, constipation, etc.)	45	0.83–15	25 (55.56)	NR	NR	3/45 (6.67)	2/45 (4.44)	NR	Chinese
Chen (2020) [34]	South	Guangdong	tTG‐IgA, DGP‐IgA, DGP‐IgG	T1DM, RA, AS, IBS, colitis, diarrhea, anemia, low BMI, psoriasis, short stature, Bristol Stool Scale 6 or 7	711	≥ 18	446 (62.73)	9/711 (1.26)	1/711 (0.14) 1/9 (11.11)	NR	DGP‐IgA: 4/711 (0.56) 4/9 (44.44) DGP‐IgG: 4/711 (0.56) 4/9 (44.44)	NR	English
Guo (2016) [35]	East	Jiangsu	tTG‐IgA, biopsy	GI symptoms^a^ (diarrhea, nausea or vomiting, abdominal pain or distension, constipation), weight loss, Down's syndrome, developmental delay, T1DM	140	0.5–13	77 (55.00)	NR	7/140 (5.00)	NR	NR	Performed in three patients (3/3 [100])	Chinese
Jiang (2009) [36]	East	Zhejiang	Biopsy	GI symptoms (diarrhea)	62	19–75	42 (67.74)	4/62 (6.45)	NR	NR	NR	4/62 (6.45)	English
Kang (2018) [37]	Central	Henan	AGA, tTG‐IgA, DGP‐IgA/IgG, biopsy	GI symptoms (diarrhea)	756	NR	NR	44/756 (5.82)	NR	NR	NR	44/756 (5.82)	Chinese
Kong (2016) [38]	North	Beijing	tTG‐IgA/IgG, EMA‐IgA/IgG, AGA‐IgA/IgG, biopsy	GI symptoms (abdominal pain, distension, vomiting, diarrhea, constipation, bloody stool)	162	0.67–13	97 (59.88)	11/162 (6.79)	11/162 (6.79)	10/162 (6.17)	11/162 (6.79)	7/162 (4.32)	Chinese
Kou (2013) [39]	East	Shandong	tTG‐IgA, biopsy	IBS	126	19–73	60 (47.62)	3/126 (2.38)	10/126 (7.94)	NR	NR	Performed in 3 patients 3/3 (100)	Chinese
Kou (2018) [18]	East	Shandong	tTG‐IgA, biopsy	IBS	246	18–74	115 (46.75)	7/246 (2.85)	12/246 (4.88)	NR	NR	Performed in 5 patients 5/5 (100)	English
Li (2010) [40]	North	Beijing	tTG‐IgA, EMA, AGA, biopsy	GI symptoms (diarrhea, abdominal distension), malnutrition, infertility, decreased bone mineral density, dermatitis herpetiformis	147	0.67–60	NR	28/147 (19.05)	9/147 (6.12) 5/9 (55.56)	IgA: 9/147 (6.12) 5/9 (55.56) IgG: 2/147 (1.36) 1/2 (50.0)	IgA: 21/147 (14.29) 13/21 (61.90) IgG: 36/147 (24.49) 16/36 (44.44)	10/18 (55.56)	Chinese
Liao (2020) [41]	North	Beijing	tTG‐IgA/IgG, AGA‐IgA/IgG, biopsy	GI symptoms (abdominal pain, diarrhea, vomiting, nausea)	116	0.5–15	69 (59.48)	2/113 (1.77)	IgA: 1/116 (0.86)	NR	IgA: 7/116 (6.03) IgG: 2/116 (1.72)	2/116 (1.72)	Chinese
Liu (2017) [42]	North	Tianjin	tTG‐IgA, biopsy	IBS	136	20–76	85 (62.50)	3/136 (2.21)	5/136 (3.68)	NR	NR	3/5 (60.0)	Chinese
Liu (2021) [43]	South	Guangdong	tTG‐IgA, DGP‐IgA/IgG	IBS^a^, colitis, diarrhea^a^, anemia, low BMI, short stature, T1DM^a^, RA, AS, psoriasis, Bristol Stool Scale 6 or 7	1273	19–97	491 (38.57)	12/1273 (0.94)	1/1273 (0.08)	NR	IgA: 6/1273 (0.47) IgG: 5/1273 (0.39)	NR	Chinese
Lyu (2010) [44]	East	Jiangsu	tTG‐IgA, AGA‐IgG	GI symptoms	73	18–80	NR	6/73 (8.22) Seroprevalence	2/73 (2.74)	NR	IgG: 5/73 (6.85)	NR	Chinese
Ma (2014) [45]	North	Beijing	tTG‐IgA/IgG	IBD or chronic diarrhea	227	NR	122 (53.74)	12/227 (5.29) Seroprevalence	IgA: 5/227 (2.20) IgG: 7/227 (3.08)	NR	NR	NR	Chinese
Pan (2006) [19]	East	Anhui	EMA‐IgA/IgG	GI symptoms (diarrhea, hemorrhage, abdominal pain, tumor, constipation)	136	3–18	73 (53.68)	Significant positivity 2/136 (1.47)	NR	IgA: 19/136 (13.97) IgG: 16/136 (11.76)	NR	NR	Chinese
Shang (2021) [46]	East	Shandong	tTG‐IgA, EMA‐IgA	T1DM	76	3–18	40 (52.63)	6/76 (7.89)	4/76 (5.26)	IgA: 3/76 (3.95)	NR	NR	Chinese
Wang (2009/2010/2011^b^) [47, 48, 49]	East, Central, Southwest	Shanghai, Shandong, Hubei, Sichuan	tTG‐IgA, EMA, biopsy	GI symptoms (diarrhea)	118	0–18	85 (72.03)	14/118 (11.86)	27/118 (22.88)	19/118 (16.10)	NR	14/18 (77.78)	Chinese/English
Wang (2015) [17]	Central	Hubei	tTG/DGP‐IgA/IgG, biopsy	IBS	395	18–80	183 (46.33)	4/395 (1.01)	7/395 (1.77)	NR	7/395 (1.77)	4/7 (57.14)	English
Li/Zhou/Wang/Liu (2020/2021/2022^b^/2023) [50, 51, 52, 53]	Northwest	Xinjiang	tTG‐IgA, biopsy	GI symptoms (diarrhea, abdominal pain or distension, constipation, vomiting, nausea, anorexia, heartburn, acid reflux, burping)	2884	2–96	1531 (53.09)	50/2884 (1.73)	73/2884 (2.53)	NR	NR	50/69 (72.46)	Chinese/English
Wu/Zhao (2010/2010^b^/2013) [54, 55, 56]	East	Jiangsu	tTG‐IgA, AGA‐IgG	IBS	73	NR	45 (61.64)	7/73 (9.59)	2/73 (2.74)	NR	5 (6.85)	NR	Chinese/English
Yuan (2015) [57]	East	Jiangxi	tTG‐IgA	Elevated transaminases	125	17–21	112 (89.60)	0/125 (0)	0/125 (0)	NR	NR	NR	English
Zhang (2016) [58]	Central	Henan	tTG‐Ab, DGP‐Ab	Non‐segmental vitiligo	165	2–59	97 (58.79)	NR	6/165 (3.64)	NR	18/165 (10.91)	NR	Chinese
Zhang (2022) [59]	East	Shanghai	tTG‐IgA/IgG, EMA‐IgA/IgG, DGP‐IgA/IgG	T1DM	80	≤ 18	41 (51.25)	28/80 (35.00)	IgA: 2/80 (2.50) IgG: 0/80 (0)	IgA: 8/80 (10.0) IgG: 19/80 (23.75)	DGP‐IgA: NR DGP‐IgG: 4/80 (5.00)	NR	English
Zhang (2024) [60]	East	Shandong	tTG‐IgA, EMA‐IgA	T1DM	98	1.26–16.97	53 (54.08)	8/98 (8.16)	6/98 (6.12)	6/98 (6.12)	NR	NR	Chinese
Zhao (2016) [61]	Northeast	Jilin	tTG‐Ab	T1DM^a^, T2DM, AITD, NAITD, APS3v	503	NR	196 (38.97)	60/503 (11.93)	60/503 (11.93)	NR	NR	NR	English
Zhou (2019^b^/2020) [62, 63]	Northeast	Liaoning	tTG‐Ab, EMA, AGA	GI and extra‐GI symptoms	152	0.5–15	83 (54.61)	27/152 (17.76) seroprevalence 7/152 (4.61) sero+gene	4/152 (2.63)	19/152 (12.50)	4/152 (2.63)	NR	Chinese
Zou (2017) [64]	East/Northeast	Jiangsu/Jilin	tTG‐Ab	T1DM^a^, T2DM	308	2–72	142 (46.10)	29/308 (9.42)	29/308 (9.42)	NR	NR	NR	Chinese

Abbreviations: AGA, anti‐gliadin antibody; AITD, autoimmune thyroid disease; APS3v, autoimmune polyglandular syndrome type 3 variant; AS, ankylosing spondylitis; BMI, body mass index; DGP, deamidated gliadin antibody; EMA, anti‐endomysial antibody; GI, gastrointestinal; IBS, irritable bowel syndrome; NA, not applicable; NAITD, non‐autoimmune thyroid disease; NR, not reported; RA, rheumatoid arthritis; SD, standard deviation; anti‐tTG Ab, anti‐tissue transglutaminase antibody; T1DM, type 1 diabetes mellitus; T2DM, type 2 diabetes mellitus.

^a^ The article included patients with multiple diseases, but also reported data for the subgroup of IBS, T1DM or GI symptoms.

^b^ When a study includes multiple reports, the primary study is marked with a # symbol.

^c^ The CeD positivity rate is finally established in the article based on seropositivity or histological confirmation.

Evaluation of the 28 studies revealed a spectrum of quality, with notable strengths including sample frame appropriateness, sampling method adequacy, and robust identification methodologies, which were identified in all studies, as well as standardized measurement techniques and rigorous statistical analysis. The areas for improvement included sample size adequacy, as only 71.43% (*n* = 20) of the studies were assessed as having a sufficient sample size (Table S2).

### 3.1 Prevalence of Biopsy‐Confirmed CeD Among High‐Risk Populations

Thirteen studies (18 references) [17, 18, 32, 35, 36, 37, 38, 39, 40, 41, 42, 47, 48, 49, 50, 51, 52, 53], involving 5486 patients, examined the prevalence of CeD among high‐risk populations based on a biopsy‐confirmed finding. The meta‐analysis revealed a biopsy‐confirmed CeD prevalence of 3.69% (95% CI 2.32%–5.37%; Figure S1). The heterogeneity was high (84%).

#### 3.1.1 Prevalence of Biopsy‐Confirmed CeD Among High‐Risk Populations Across Different Regions of China

**FIGURE 2:**
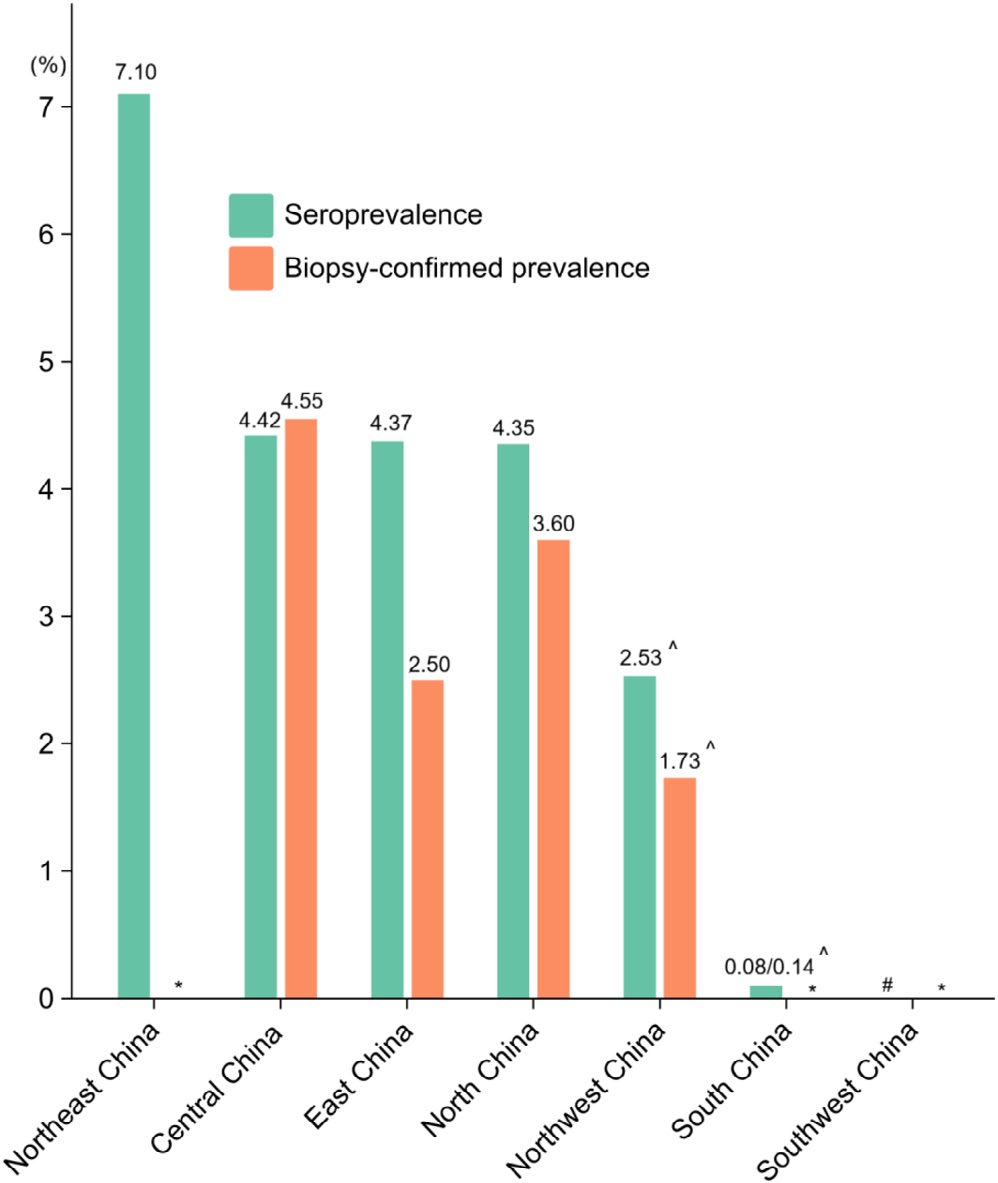
Seroprevalence and biopsy‐confirmed prevalence of celiac disease (CeD) in high‐risk populations across different regions of China. Seroprevalence was determined by positive anti‐tissue transglutaminase antibody and/or anti‐endomysium antibody. *No studies reported the biopsy‐confirmed prevalence of CeD in Northeast, South, or Southwest China. ^#^No study reported the seroprevalence of CeD in Southwest China. ^^^Data were not pooled since there were fewer than three studies.

Twelve studies (15 references) [17, 18, 32, 35, 36, 37, 38, 39, 40, 41, 42, 50, 51, 52, 53], involving 5368 patients, reported the biopsy‐confirmed CeD prevalence among high‐risk populations across different regions of China (Figure 2). The pooled prevalence was 4.55% (95% CI 0.66%–8.44%) in Central China (three studies [17, 32, 37] involving 1349 patients), 2.50% (95% CI 1.39%–3.94%) in East China (four studies [18, 35, 36, 39] with 574 patients), 3.60% (95% CI 1.75%–6.08%) in North China (four studies [38, 40, 41, 42] with 561 patients), and 1.73% in Northwest China (one study [four references] [50, 51, 52, 53] including 2884 patients). No studies reported the biopsy‐confirmed prevalence of CeD in Northeast, South, or Southwest China. Accounting for regional population sizes [30] using Equation (1), the total prevalence of biopsy‐confirmed CeD among the high‐risk populations in China was estimated to be 3.27% (95% CI 2.10%–4.44%).

#### 3.1.2 Prevalence of Biopsy‐Confirmed CeD Among High‐Risk Populations Regarding Risk Factors

**FIGURE 3:**
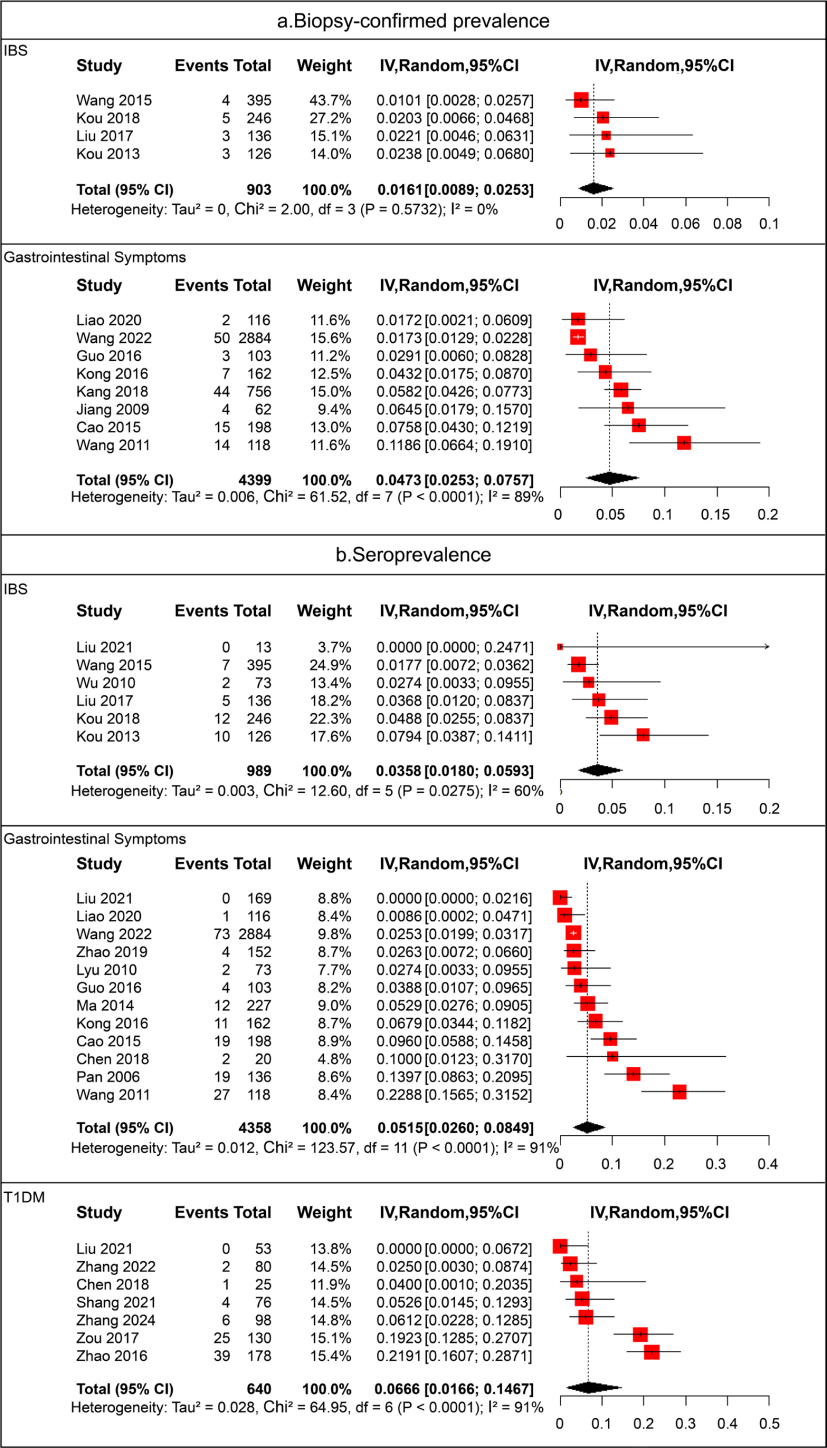
Forest plots of (a) biopsy‐confirmed prevalence and (b) seroprevalence of celiac disease in Chinese high‐risk populations with irritable bowel syndrome (IBS), type 1 diabetes mellitus (T1DM), or gastrointestinal symptoms.

Four studies [17, 18, 39, 42] involving 903 patients focused on CeD in those with IBS. The pooled prevalence of CeD in patients with IBS was 1.61% (95% CI 0.89%–2.53%), while eight studies (13 references) [32, 35, 36, 37, 38, 41, 47, 48, 49, 50, 51, 52, 53] (including 4399 patients) investigated patients with GI symptoms, for which the pooled prevalence was 4.73% (95% CI 2.53%–7.57%) (Figure 3a). Moreover, the prevalence of biopsy‐confirmed CeD in adult and pediatric patients among the high‐risk populations ranged between 1.61% and 4.63%, as shown in Table S3.

### 3.2 Seroprevalence of CeD Among High‐Risk Populations

Altogether 26 studies (34 references) [17, 18, 19, 32, 33, 34, 35, 38, 39, 40, 41, 42, 43, 44, 45, 46, 47, 48, 49, 50, 51, 52, 53, 54, 55, 56, 57, 58, 59, 60, 61, 62, 63, 64] involving 8676 patients reported the tTG‐Ab/EMA‐positive rate among the high‐risk populations, indicating a pooled seroprevalence rate of 4.43% (95% CI 2.75%–6.49%) (Figure S2).

#### 3.2.1 Seroprevalence of CeD Among High‐Risk Populations Across Different Regions of China

The rate of tTG‐Ab/EMA seropositivity was observed to vary across different regions in China, based on 24 studies (30 references) [17, 18, 19, 32, 33, 34, 35, 38, 39, 40, 41, 42, 43, 44, 45, 46, 50, 51, 52, 53, 54, 55, 56, 57, 58, 59, 60, 61, 62, 63] with 8287 patients (Figure 2). In Central China (three studies [17, 32, 58] including 758 patients), the pooled rate was found to be 4.42% (95% CI 1.04%–9.98%). In East China (10 studies (12 references) [18, 19, 35, 39, 44, 46, 54, 55, 56, 57, 59, 60] involving 1173 patients), it was found to be 4.37% (95% CI 2.12%–7.38%). The seroprevalence in North China (five studies [38, 40, 41, 42, 45] with 788 patients), Northeast China (three studies [4 references] [33, 61, 62, 63] with 700 patients), and Northwest China (one study [4 references] [50, 51, 52, 53] with 2884 patients) was reported to be 4.35% (95% CI 2.39%–6.86%), 7.10% (95% CI 1.14%–13.05%), and 2.53%, respectively. In addition, two studies [34, 43] involving 1984 patients reported seroprevalence of 0.08% and 0.14% for South China. No studies were identified reporting the seroprevalence in Southwest China. When adjusted for regional population size, the seroprevalence of CeD was 4.66% (95% CI 3.04%–6.29%).

#### 3.2.2 Seroprevalence of CeD Among High‐Risk Populations Regarding Risk Factors

Seven studies [33, 43, 46, 59, 60, 61, 64] including 640 patients focused on patients with T1DM, with a pooled seroprevalence of 6.66% (95% CI 1.66%–14.67%), while six studies (eight references) [17, 18, 39, 42, 43, 54, 55, 56] (with 989 patients) reported the seroprevalence of patients with IBS, resulting in a pooled rate of 3.58% (95% CI 1.80%–5.93%). Twelve studies (18 references) [19, 32, 33, 35, 38, 41, 43, 44, 45, 47, 48, 49, 50, 51, 52, 53, 62, 63] investigated 4358 patients with GI symptoms, of which the pooled seroprevalence rate was 5.15% (95% CI 2.60%–8.49%) (Figure 3b). In addition, one study [58] involving 165 patients with non‐segmental vitiligo reported a seroprevalence rate of 3.64%. Moreover, the seroprevalence of CeD in adults and children among the high‐risk populations ranged between 3.05% and 8.13% (Table S3).

### 3.3 Heterogeneity and Publication Bias

As the majority of meta‐analyses revealed an *I* ^2^ over 50%, showing substantial heterogeneity, subgroup analyses were performed to explore its sources. However, the subgroup analyses failed to reduce the *I* ^2^ value or identify potential sources of heterogeneity. Leave‐one‐out sensitivity analysis also identified no source of heterogeneity (Figure S3). Funnel plots indicated that there was no significant publication bias regarding CeD prevalence among the high‐risk populations (Figure S4).

## 4 Discussion

In this systematic review and meta‐analysis including 28 studies comprising 9531 participants, we found that the biopsy‐confirmed prevalence and seroprevalence of CeD in China among the high‐risk populations were 3.69% and 4.43%, respectively.

Our study evaluated the prevalence of CeD in China based on the seven regions to better understand the geographical variations of the disease. Regarding regional disparities, the highest biopsy‐confirmed prevalence was observed in Central China, followed by North China, East China, and Northwest China; while for seroprevalence, the highest rate was found in Northeast China, followed by Central China, East China, North China, Northwest China, and South China. This regional disparity in CeD prevalence could potentially be due to the dietary patterns. Historically, the primary staple food of populations in Northwest China has revolved around wheat products such as noodles and steamed bread. Wheat consumption is also common in Central China; while in South China, steamed rice plays the central role [65]. Prolonged gluten exposure among people in North China may thus be associated with a higher risk of developing CeD [66].

As a multifactorial condition, regional differences in CeD prevalence within China likely reflect complex interactions among genetic, environmental, dietary, and healthcare factors. For example, HLA‐DQ2 and DQ8 antigens, which are key genetic risk alleles for CeD, are not uniformly distributed across Chinese populations, while higher frequencies are reported in North and Central regions [21]. Environmental exposure, including early‐life infections and gut microbiota composition, may also differ regionally because of sanitation levels, climate, and antibiotic use patterns [67]. In Northeast China, a colder climate, increased early‐life infections, and more frequent antibiotic use may have contributed to altered gut microbiota and increased CeD seropositivity. Furthermore, disparities in healthcare access and diagnostic capacity across urban–rural and regional divisions in China are well documented. East and Central China typically benefit from more advanced medical infrastructure and higher levels of clinician awareness, potentially leading to higher rates of CeD detection through serological testing and biopsy confirmation. In contrast, cases with CeD are underdiagnosed in Western China and rural areas because of limited awareness, lower health‐seeking behavior, and inadequate gastroenterology specialists [68]. In addition, serological test availability and quality may vary by region, affecting the measured seroprevalence. Therefore, interpreting regional CeD prevalence requires a comprehensive framework that accounts for dietary patterns, genetic susceptibility, healthcare accessibility, and population‐level awareness specific to China.

Regarding the prevalence of CeD among the high‐risk populations, our biopsy‐confirmed prevalence of 3.69% (95% CI 2.32%–5.37%) was consistent with previously reported estimates of 4.3% (95% CI 3.3%–5.5%) in the Asia‐Pacific region [69] and 4.44% (95% CI 1.53%–8.58%) in China [14]. Specifically, patients with GI symptoms exhibited a higher biopsy‐confirmed rate than those with IBS. For CeD seroprevalence, the rate was highest among patients with T1DM, followed by those with GI symptoms and those with IBS. Specifically, our results revealed that biopsy‐confirmed prevalence of CeD among patients with GI symptoms in China was higher than the global rate (4.73% vs. 1.5%) [70], whereas its seroprevalence was comparable to the global figure (5.15% vs. 4.8%) [70]. In addition, we observed a lower seroprevalence among T1DM patients in China compared with a population from Saudi Arabia (6.66% vs. 15.88%) [71]. We also found a lower seroprevalence among the high‐risk populations than that in Zhou et al.'s study (4.43% vs. 8.34%) [14]. These findings may be partly explained by differences among the included studies and the various characteristics of the enrolled high‐risk patients. Our meta‐analysis included 28 studies over an extended time frame. Notably, our results may be predominantly influenced by the rates observed in patients with GI symptoms (i.e., 5.15% and 95% CI 2.60%–8.49% based on 12 studies with 4358 patients). The inclusion of a more extensive range of studies in our review may provide a more comprehensive representation of high‐risk populations.

There were some limitations to our study, which primarily reflect the challenges of synthesizing evidence on CeD prevalence in China. Our meta‐analysis showed significant heterogeneity, which remained unexplained despite predefined subgroup analyses. Factors such as the year of publication and sample size might have contributed to this variability. In addition, although subgroup analyses were conducted, including those targeting different age groups (children and adults) and high‐risk conditions, they did not provide insights into the underlying sources of heterogeneity. Moreover, although we carefully selected and analyzed the data using rigorous methods, publication bias remained a potential source of heterogeneity. While we specified that the diagnostic criteria for including studies on CeD required the use of serology (tTG‐Ab or EMA) or biopsy, different studies might have utilized varied serological assays and cut‐off values according to the instructions of the manufacturers. This could also be a potential source of heterogeneity.

One critical observation of our study is the limited clinical awareness of CeD in China, likely contributing to the scarcity of high‐quality research in this area. This is evidenced by the predominance of Chinese‐language retrospective studies in our dataset, many of which lacked detailed diagnostic criteria or clear reporting of methods. Differences in serology assay kits, diagnostic thresholds, and biopsy practices across studies further exacerbated the heterogeneity. The overall quality of the included studies was variable, adding another layer of complexity to the observed heterogeneity.

One notable knowledge gap identified in our study is the limited availability of references providing estimates of CeD prevalence in the general population of China. A comprehensive search with rigorous inclusion criteria identified only two studies reporting general seroprevalence rates of 0.36% (among 19 778 patients) [15] and 0.03% (among 8794 pediatric patients) [20]. Further research is crucial to fill this gap and establish healthcare policies and strategies aimed at improving the diagnosis, management, and prevention of CeD in the Chinese population.

## 5 Conclusions

We found a relatively high prevalence of CeD in China among patients with high‐risk conditions such as GI symptoms, IBS, and T1DM. Specifically, the pooled biopsy‐confirmed CeD prevalence among these high‐risk populations was 3.69%, while the seroprevalence was slightly higher at 4.43%. Moreover, the prevalence of CeD showed regional discrepancies among different regions of China, with a higher prevalence in North China than in South China. However, these findings should be interpreted with caution given the scarcity of population‐based prevalence studies, underscoring the urgent need for further research in this area to obtain more accurate estimates of CeD prevalence in China.

## Conflicts of Interest

Min Hu Chen is the co‐Editor‐in‐Chief for the *Journal of Digestive Diseases* but was not involved in the editorial review or the decision to publish this article. Min Hu Chen was a consultant for advisory functions at I‐Mab Biopharma and received speaker honoraria from Xi'an Janssen, AbbVie, AstraZeneca China, Ipsen Tianjin, Takeda China, and CMS China. Ying Lian Xiao declares no conflicts of interest. Ya Qi Jia is an employee of Takeda (China) Holdings Co. Ltd. and Li Qun Gu is an employee of Takeda Development Center Asia.

## Supporting information


**Figure S1:** Forest plot of biopsy‐confirmed prevalence of celiac disease in high‐risk populations. CI, confidence interval.

**Figure S2:** Forest plot of seroprevalence of celiac disease in high‐risk populations.

**Figure S3:** Results of leave‐one‐out sensitivity analysis for (a) biopsy‐confirmed prevalence and (b) seroprevalence of celiac disease among high‐risk populations in China.

**Figure S4:** Funnel plot for (a) biopsy‐confirmed prevalence and (b) seroprevalence of celiac disease among high‐risk populations in China.

**Data S1:** Supplementary Tables.

## Data Availability

The data that support the findings of this study are available from the corresponding author upon reasonable request.
