# Supplementary figures and images for: Prevalence of Celiac Disease in China Among High‐Risk Populations: A Systematic Review and Meta‐Analysis
Source: J Dig Dis. 2025 Nov 3;26(9-10):414–27. doi: 10.1111/1751-2980.70013 (PMC12681390; doi:10.1111/1751-2980.70013)

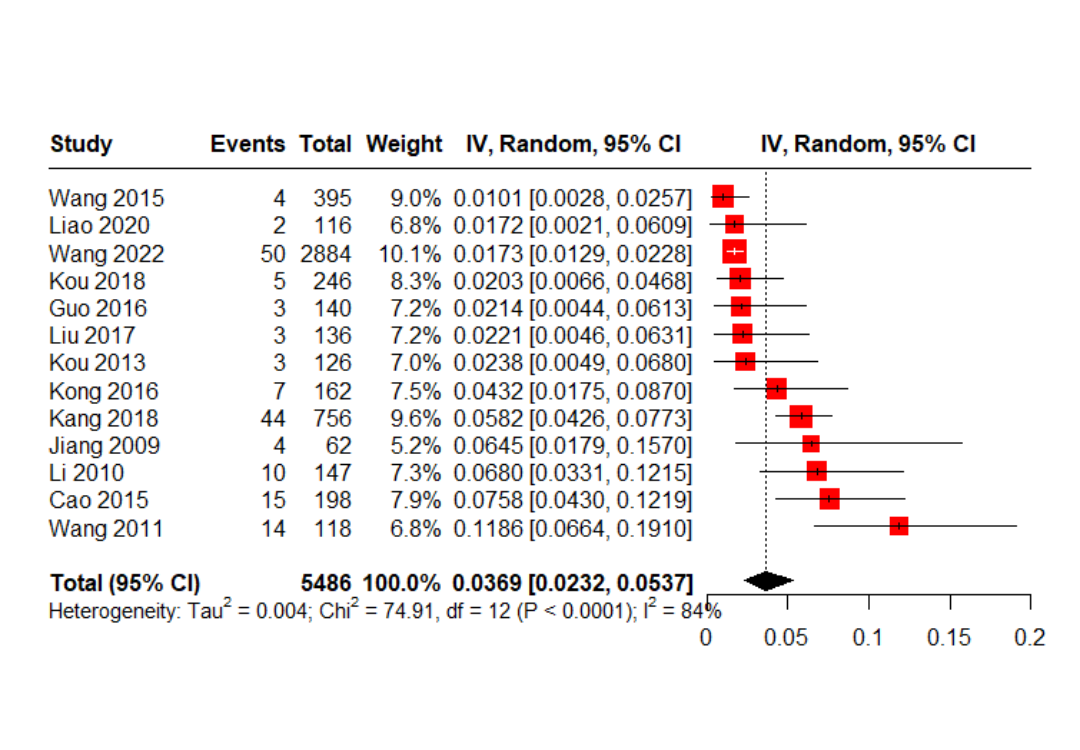

Supplement: Supplementary file 1 — Figure S1: Forest plot of biopsy‐confirmed prevalence of celiac disease in high‐risk populations. CI, confidence interval. [file CDD-26-414-s005.tiff]

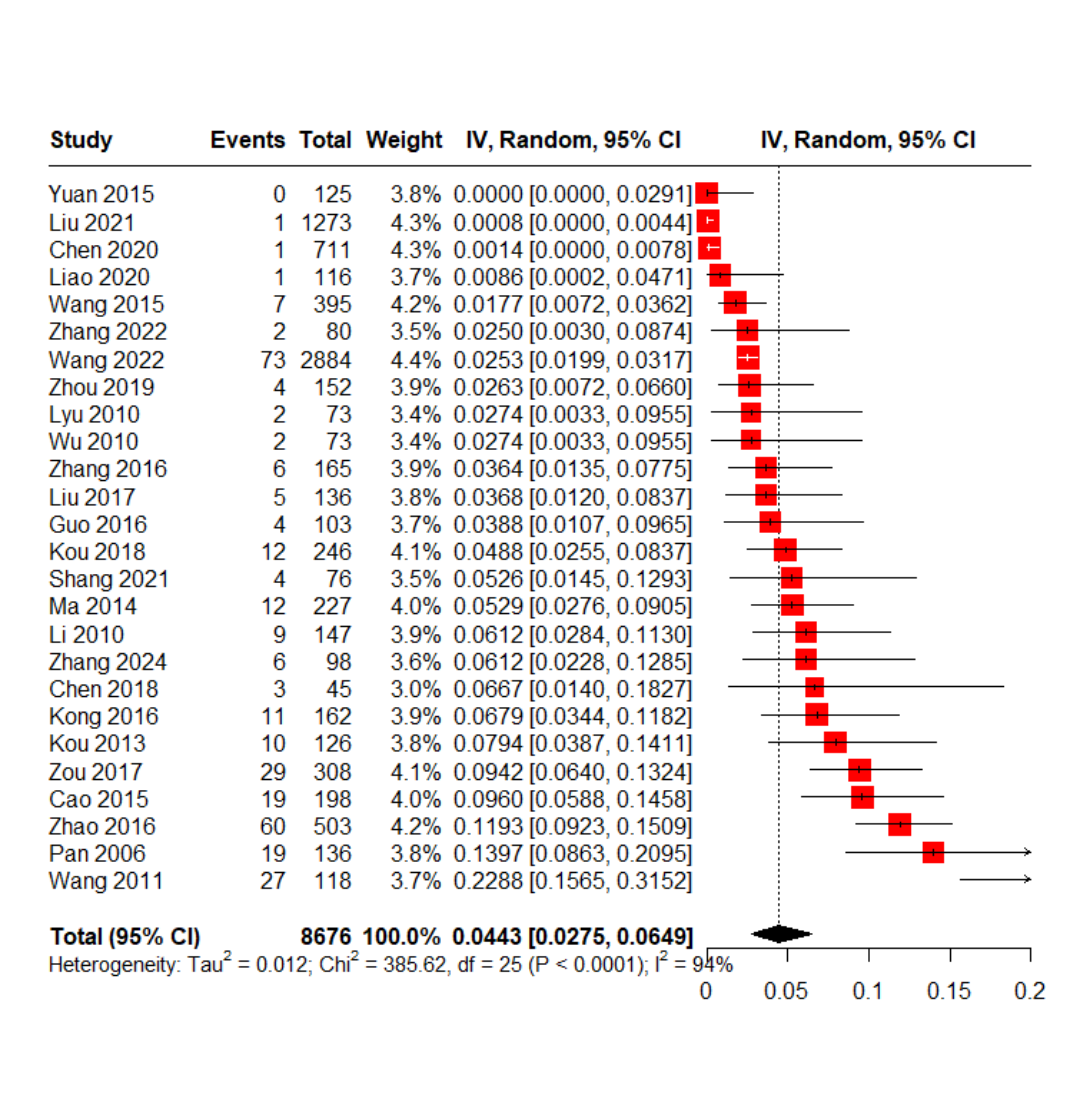

Supplement: Supplementary file 2 — Figure S2: Forest plot of seroprevalence of celiac disease in high‐risk populations. [file CDD-26-414-s001.tiff]

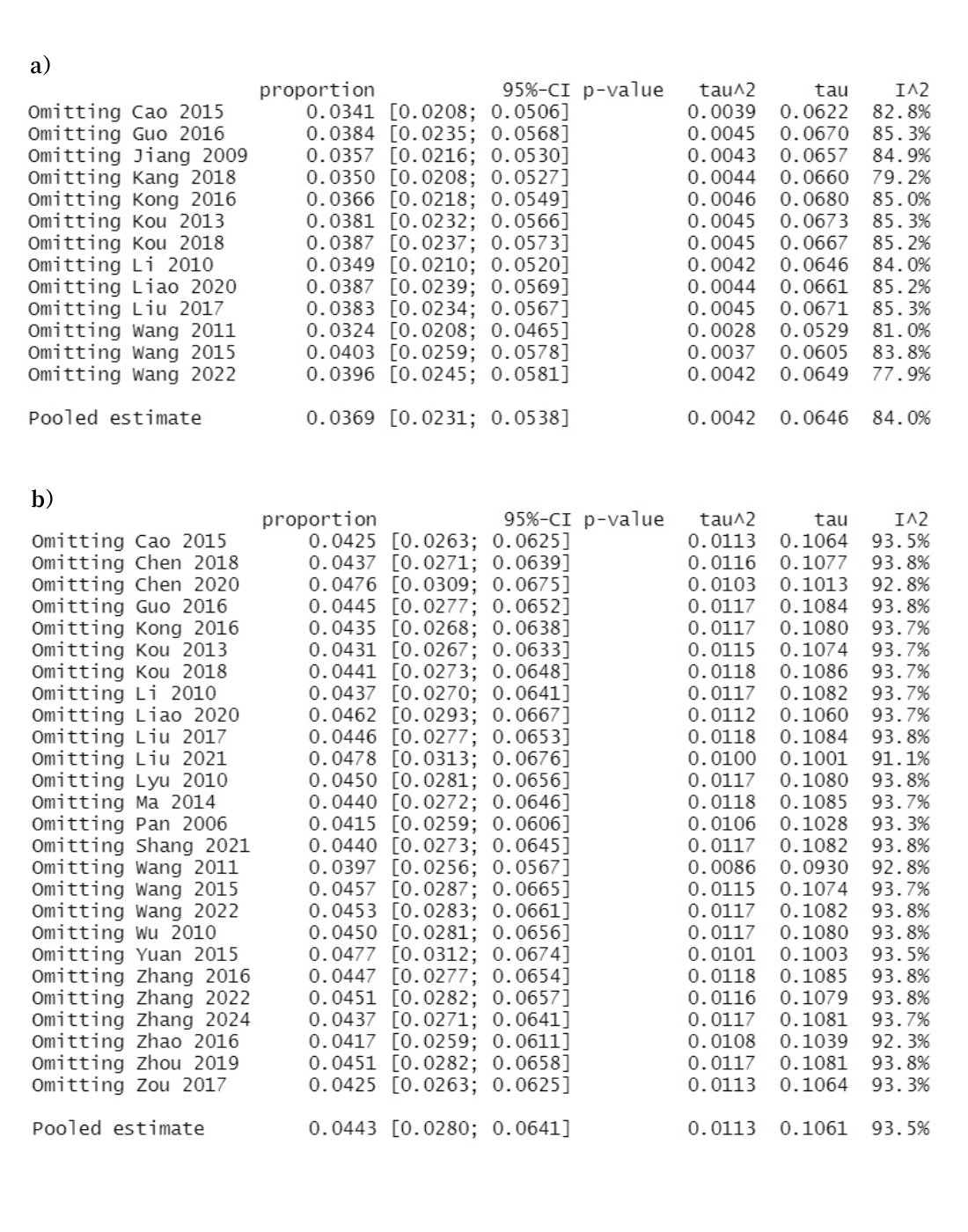

Supplement: Supplementary file 3 — Figure S3: Results of leave‐one‐out sensitivity analysis for (a) biopsy‐confirmed prevalence and (b) seroprevalence of celiac disease among high‐risk populations in China. [file CDD-26-414-s003.tiff]

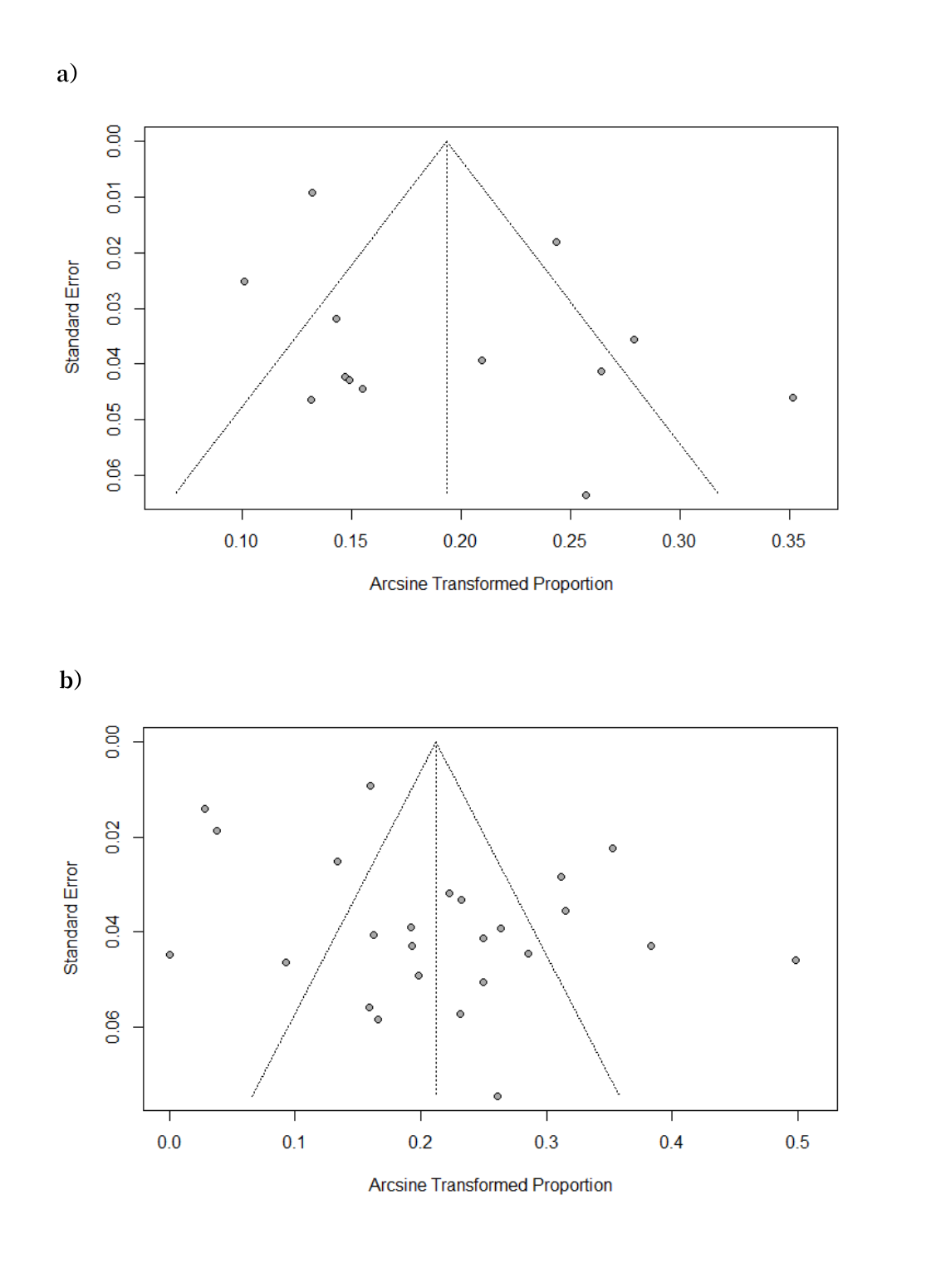

Supplement: Supplementary file 4 — Figure S4: Funnel plot for (a) biopsy‐confirmed prevalence and (b) seroprevalence of celiac disease among high‐risk populations in China. [file CDD-26-414-s002.tiff]
