# Supplementary material for: Prevalence of Celiac Disease in China Among High‐Risk Populations: A Systematic Review and Meta‐Analysis
Source: J Dig Dis. 2025 Nov 3;26(9-10):414–27. doi: 10.1111/1751-2980.70013 (PMC12681390; doi:10.1111/1751-2980.70013)
Supplement: Supplementary file 5 — Data S1: Supplementary Tables. [file CDD-26-414-s004.docx]

# Table S1. Search strategies for literature search in different databases.

| **EMBASE** #1. 'celiac disease'/exp OR (((coeliac OR celiac OR coeliaky OR nontropical OR "gee herter" OR "gee thaysen" OR "huebner herter") NEAR/3 (disease* OR Sprue OR syndrome*)) OR (Gluten NEAR/3 (Enteropath* OR intoleran*))):ab,ti,kw  #2. 'China'/exp OR (China OR Chinese OR Taiwan OR "Hong kong" OR Hongkong OR Macau OR Macao OR Beijing OR Shanghai OR Tianjin OR Chongqing OR "Inner Mongolia" OR Tibet OR Guangxi OR Sinkiang OR Ningxia OR Xinjiang OR Hebei OR Shanxi OR Liaoning OR Jilin OR Heilongjiang OR Jiangsu OR Zhejiang OR Anhui OR Fujian OR Jiangxi OR Shandong OR Henan OR Hubei OR Hunan OR Guangdong OR Hainan OR Sichuan OR Guizhou OR Yunnan OR Shaanxi OR Gansu OR Qinghai):ti,ab,ad,ff  #3 #1 and #2 |
| --- |
| **PubMed** #1 'celiac disease'/exp 21605  #2 (((coeliac OR celiac OR coeliaky OR nontropical OR "gee herter" OR "gee thaysen" OR "huebner herter") and (disease* OR Sprue OR syndrome*)) OR (Gluten and (Enteropath* OR intoleran*)))  #3 #1 or #2  #4 China/exp  #5 (China OR Chinese OR Taiwan OR "Hong kong" OR Hongkong OR Macau OR Macao OR Beijing OR Shanghai OR Tianjin OR Chongqing OR "Inner Mongolia" OR Tibet OR Guangxi OR Sinkiang OR Ningxia OR Xinjiang OR Hebei OR Shanxi OR Liaoning OR Jilin OR Heilongjiang OR Jiangsu OR Zhejiang OR Anhui OR Fujian OR Jiangxi OR Shandong OR Henan OR Hubei OR Hunan OR Guangdong OR Hainan OR Sichuan OR Guizhou OR Yunnan OR Shaanxi OR Gansu OR Qinghai)  #6 #4 OR #5  #7 #3 and #6 |
| **Cochrane Library**  #1 'celiac disease'/exp  #2 (((coeliac OR celiac OR coeliaky OR nontropical OR "gee herter" OR "gee thaysen" OR "huebner herter") and (disease* OR Sprue OR syndrome*)) OR (Gluten and (Enteropath* OR intoleran*)))  #3 #1 or #2  #4 China/exp  #5 (China OR Chinese OR Taiwan OR "Hong kong" OR Hongkong OR Macau OR Macao OR Beijing OR Shanghai OR Tianjin OR Chongqing OR "Inner Mongolia" OR Tibet OR Guangxi OR Sinkiang OR Ningxia OR Xinjiang OR Hebei OR Shanxi OR Liaoning OR Jilin OR Heilongjiang OR Jiangsu OR Zhejiang OR Anhui OR Fujian OR Jiangxi OR Shandong OR Henan OR Hubei OR Hunan OR Guangdong OR Hainan OR Sichuan OR Guizhou OR Yunnan OR Shaanxi OR Gansu OR Qinghai)  #6 #4 OR #5  #7 #3 and #6 |
| **Web of Science** #1 celiac disease or (((coeliac OR celiac OR coeliaky OR nontropical OR "gee herter" OR "gee thaysen" OR "huebner herter") and (disease* OR Sprue OR syndrome*)) OR (Gluten and (Enteropath* OR intoleran*)))  #2 ((China OR Chinese OR Taiwan OR "Hong kong" OR Hongkong OR Macau OR Macao OR Beijing OR Shanghai OR Tianjin OR Chongqing OR "Inner Mongolia" OR Tibet OR Guangxi OR Sinkiang OR Ningxia OR Xinjiang OR Hebei OR Shanxi OR Liaoning OR Jilin OR Heilongjiang OR Jiangsu OR Zhejiang OR Anhui OR Fujian OR Jiangxi OR Shandong OR Henan OR Hubei OR Hunan OR Guangdong OR Hainan OR Sichuan OR Guizhou OR Yunnan OR Shaanxi OR Gansu OR Qinghai))  #3 #1 and #2 |
| **China National Knowledge Infrastructure (CNKI)** TKA = '乳糜泻' OR TKA = '谷胶病' OR TKA = '特发性脂肪泻' OR TKA = '麸质肠病' OR TKA = '麸胶敏感性肠病' OR TKA = '口炎性脂肪泻' OR SU %= '乳糜泻' OR SU %= '谷胶病' OR SU %= ''特发性脂肪泻' OR SU %= '麸质肠病' OR SU %= '麸胶敏感性肠病' OR SU %= '口炎性脂肪泻' |
| **Wanfang Digital Resource Database (Wanfang)** 全部:("乳糜泻") or 全部:("谷胶病") or 全部:("特发性脂肪泻") or 全部:("麸质肠病") or 全部:("麸胶敏感性肠病") or 全部:("口炎性脂肪泻") |
| **VIP’s Chinese Science and Technology Journal Database (VIP)** U="乳糜泻" or "谷胶病" or "特发性脂肪泻" OR "麸质肠病" OR "麸胶敏感性肠病" OR "口炎性脂肪泻" |
| **Chinese Biomedical Database (CBM)** "乳糜泻"[不加权: 扩展] or "谷胶病"[常用字段:智能] or "乳糜泻"[常用字段:智能] or "特发性脂肪泻"[常用字段:智能] OR "麸质肠病"[常用字段:智能] OR "麸胶敏感性肠病"[常用字段:智能] OR "口炎性脂肪泻"[常用字段:智能] |

# Table S2. Quality assessment of prevalence studies based on Joanna Briggs Institute (JBI) critical appraisal checklist for prevalence studies

| **Study no.** | **First author (year of publication)** | **Questions assessing the cross–sectional studies** | | | | | | | | |
| --- | --- | --- | --- | --- | --- | --- | --- | --- | --- | --- |
|  |  | 1 | 2 | 3 | 4 | 5 | 6 | 7 | 8 | 9 |
| 1 | Cao (2015) [32] | Y | Y | Y | Y | Y | Y | Y | Y | Y |
| 2 | Chen (2018) [33] | Y | Y | N | Y | Y | Y | Y | Y | Y |
| 3 | Chen (2020) [34] | Y | Y | Y | Y | Y | Y | Y | Y | Y |
| 4 | Guo (2016) [35] | Y | Y | Y | Y | Y | Y | Y | Y | Y |
| 5 | Jiang (2009) [36] | Y | Y | N | Y | Y | Y | Y | Y | Y |
| 6 | Kang (2018) [37] | Y | Y | Y | Y | Y | Y | Y | Y | Y |
| 7 | Kong (2016) [38] | Y | Y | N | Y | Y | Y | Y | Y | Y |
| 8 | Kou (2013) [39] | Y | Y | Y | Y | Y | Y | Y | Y | Y |
| 9 | Kou (2018) [18] | Y | Y | Y | Y | Y | Y | Y | Y | Y |
| 10 | Li (2010) [40] | Y | Y | Y | Y | Y | Y | Y | Y | Y |
| 11 | Liao (2020) [41] | Y | Y | Y | Y | Y | Y | Y | Y | Y |
| 12 | Liu (2017) [42] | Y | Y | Y | Y | Y | Y | Y | Y | Y |
| 13 | Liu (2021) [43] | Y | Y | Y | Y | Y | Y | Y | Y | Y |
| 14 | Lyu (2010) [44] | Y | Y | Y | Y | Y | Y | Y | Y | Y |
| 15 | Ma (2014) [45] | Y | Y | Y | Y | Y | Y | Y | Y | Y |
| 16 | Pan (2006) [19] | Y | Y | N | Y | Y | Y | Y | Y | Y |
| 17 | Shang (2021) [46] | Y | Y | Y | Y | Y | Y | Y | Y | Y |
| 18 | Wang (2009/2010/2011^#^) [47–49] | Y | Y | N | Y | Y | Y | Y | Y | Y |
| 19 | Wang (2015) [17] | Y | Y | Y | Y | Y | Y | Y | Y | Y |
| 20 | Li/Zhou/Wang/Liu (2020/2021/2022^#^/ 2023) [50–53] | Y | Y | Y | Y | Y | Y | Y | Y | Y |
| 21 | Wu/Zhao  (2010/2010^#^/2013) [54–56] | Y | Y | Y | Y | Y | Y | Y | Y | Y |
| 22 | Yuan (2015) [57] | Y | Y | Y | Y | Y | Y | Y | Y | Y |
| 23 | Zhang (2016) [58] | Y | Y | Y | Y | Y | Y | Y | Y | Y |
| 24 | Zhang (2022) [59] | Y | Y | N | Y | Y | Y | Y | Y | Y |
| 25 | Zhang (2024) [60] | Y | Y | Y | Y | Y | Y | Y | Y | Y |
| 26 | Zhao (2016) [61] | Y | Y | N | Y | Y | Y | Y | Y | Y |
| 27 | Zhou (2019^#^/2020) [62, 63] | Y | Y | N | Y | Y | Y | Y | Y | Y |
| 28 | Zou (2017) [64] | Y | Y | Y | Y | Y | Y | Y | Y | Y |

^#^When a study includes multiple reports, the primary study is marked with a # symbol.

*Notes:* 1. Was the sample frame appropriate to address the target population? 2. Were study participants sampled in an appropriate way? 3. Was the sample size adequate? 4. Were the study subjects and the setting described in detail? 5. Was the data analysis conducted with sufficient coverage of the identified sample? 6. Were valid methods used for the identification of the condition? 7. Was the condition measured in a standard, reliable way for all participants? 8. Was there appropriate statistical analysis? 9. Was the response rate adequate, and if not, was the low response rate managed appropriately?

Abbreviations: Y, Yes; N, No; U, Unclear.

# Table S3. Meta-analysis results of seroprevalence or biopsy-confirmed prevalence of celiac disease in adults and children among Chinese high-risk populations

| **Population** | **Adult/Children** | **Antibody/Biopsy** | **Number of Studies (Sample size)** | **Outcomes (95% CI)** |
| --- | --- | --- | --- | --- |
| **High-risk** | **Adult** | **Biopsy-confirmed** | 7 (*n* = 3764) | 2.66% (1.47%–4.19%); *I*^2^ = 72% |
|  |  | **Seroprevalence** | 15 (*n* =7076) | 3.55% (1.61%–6.20%); *I*^2^ = 96% |
|  | **Children** | **Biopsy-confirmed** | 5 (*n* = 782) | 3.63% (1.13%–7.44%); *I*^2^ = 82% |
|  |  | **Seroprevalence** | 11 (*n* = 1146) | 7.39% (3.70%–12.22%); *I*^2^ = 87% |
| **IBS** | **Adult** | **Biopsy-confirmed** | 4 (*n* = 903) | 1.61% (0.89%–2.53%); *I*^2^ = 0% |
|  |  | **Seroprevalence** | 6 (*n* = 989) | 3.58% (1.80%–5.93%); *I*^2^ = 60% |
| **GI symptoms** | **Adult** | **Biopsy-confirmed** | 3 (*n* = 2861) | 4.63% (1.07%–10.50%); *I*^2^ = 89% |
|  |  | **Seroprevalence** | 5 (*n* = 3551) | 3.05% (0.84%–6.58%); *I*^2^ = 90% |
|  | **Children** | **Biopsy-confirmed** | 5 (*n* = 782) | 3.62% (1.13%–7.44%); *I*^2^ = 82% |
|  |  | **Seroprevalence** | 7 (*n* = 807) | 7.22% (2.66%–13.78%); *I*^2^ = 89% |
| **T1DM** | **Adult** | **Seroprevalence** | 3 (*n* = 301) | 8.13% (0.03%–28.20%); *I*^2^ = 95% |
|  | **Children** | **Seroprevalence** | 5 (*n* = 339) | 7.53% (2.15%–15.82%); *I*^2^ = 83% |

Abbreviations: CI, confidence interval; GI, gastrointestinal; IBS, irritable bowel syndrome; T1DM, type 1 diabetes mellitus.

**Supplementary Figure Legends**

Figure S1. Meta-analysis forest plot of biopsy-confirmed prevalence of celiac disease in high-risk populations. CI, confidence interval.

Figure S2. Meta-analysis forest plot of seroprevalence of celiac disease in high-risk populations.

Figure S3. Results of leave-one-out sensitivity analysis for (a) biopsy-confirmed prevalence and (b) seroprevalence of celiac disease among high-risk populations in China.

Figure S4. Funnel plot for meta-analysis for (a) biopsy-confirmed prevalence and (b) seroprevalence of celiac disease among high-risk populations in China.
